# αvβ6-integrin targeted PET/CT imaging in pancreatic cancer patients using ^68^Ga-Trivehexin

**DOI:** 10.3389/fnume.2024.1487602

**Published:** 2024-11-15

**Authors:** Jana Rehm, Robert Winzer, Marc Pretze, Juliane Müller, Johannes Notni, Sebastian Hempel, Marius Distler, Gunnar Folprecht, Jörg Kotzerke

**Affiliations:** ^1^ Department of Nuclear Medicine, University Hospital Dresden, Technische Universität Dresden (TUD), Dresden, Germany; ^2^ Praxis für Nuklearmedizin, Dresden, Germany; ^3^ TRIMT GmbH, Radeberg, Germany; ^4^ Department of Visceral, Thoracic and Vascular Surgery, University Hospital Dresden (TUD), Dresden, Germany; ^5^ Medical Clinic I, University Hospital Dresden (TUD), Dresden, Germany

**Keywords:** pancreatic cancer, αvβ6-integrin, positron emission tomography, radiopharmaceuticals, gallium-68

## Abstract

**Purpose:**

^68^Ga-Trivehexin is a PET tracer targeting αvβ6-integrin, a transmembrane receptor that is frequently expressed by pancreatic cancer cells. This study aimed to determine the biokinetics, image contrast, and acquisition parameters for ^68^Ga-Trivehexin PET imaging in pancreatic cancers.

**Methods:**

44 patients with pancreatic cancer underwent Trivehexin PET/CT between June 2021 and November 2022 (EK-242052023). Biokinetics and -distribution were extracted. Previous imaging follow-up imaging, and histological findings were used as reference standards. A one-way ANOVA test, followed by Tukey HSD post-hoc test was conducted. *T*-tests for subgroups ± chemotherapy prior to PET were performed. Based on dynamic PET data (*n* = 11) recorded over 45 min, time-activity curves were generated.

**Results:**

^68^Ga-Trivehexin PET/CT detected 40 pancreatic cancers, SUVmax 12.6; range [5.1–30.8]; 39 liver metastases, SUVmax 7.9 [2.7–16.3]; 21 lymph node metastases, SUVmax 8.6 [2.5–15.0]; 17 peritoneal metastases, SUVmax 9.5 [4.0–16.9] and 14 other metastases, SUVmax 7.2 [2.9–13.1]. Tukey post-hoc analysis revealed significant differences for SUVmax in pancreatic cancer compared to SUVmax in liver metastases [4.74, 95%-CI (1.74, 7.75)], for SUVmax in pancreatic cancer to SUVmax in lymph node metastasis [4.07, 95%-CI (0.47, 7. 67)], for tumor-to-liver ratio (TLR) of liver metastasis to TLR of pancreatic cancer [1.82, 95%-CI (0.83, 2.80)], for TLR of pancreatic cancer to TLR of peritoneal carcinomatoses [−1.88, 95%-CI (−3.15, −0.61)], and TLR of pancreatic cancer to TLR of pleural carcinomatosis [−2.79, 95%-CI (−5.42, −0.18)]. When comparing subgroups ± chemotherapy prior to PET, TLR of pancreatic cancers and TLR of peritoneal carcinomatoses were significantly different. At 45 min p.i., the highest tumor-to-backround (TBR) was observed.

**Conclusion:**

^68^Ga-Trivehexin is suitable for imaging of αvβ6-integrin expression in pancreatic cancer due to its ability to distinguish primary carcinoma and metastases from background tissue.

## Introduction

Pancreatic cancer is one of the deadliest malignant diseases. With a share of 8.0% it is the fourth most common cause of cancer death for both genders in Europe in 2021 (1). According to the World Health Organization's International Agency for Research on Cancer, the estimated age-standardized incidence rate (world) in 2020 for pancreatic cancer (both sexes, all ages) was 7.4% (2). A significantly worse prognosis is associated with pancreatic adenocarcinoma (PDAC). Survival rates after diagnosis are approximately 24% (1 year) and 9% (5 year). Existing diagnostic techniques lack specificity, which can lead to early-stage disease being missed and possibly not all metastases being detected (3).

The results of a comprehensive meta-analysis comparing various imaging modalities for the detection of pancreatic cancer revealed that computed tomography (CT) exhibited similar sensitivity and specificity to magnetic resonance imaging (MRI) (4). Positron emission tomography/computed tomography (PET/CT) imaging with ^18^F-FDG as a substitute for high-quality contrast-enhanced CT (ceCT) however yielded unsatisfactory results (5). Consequently, the current clinical protocols rely on ceCT and magnetic resonance imaging (MRI).

Functional imaging techniques can detect cellular changes before morphological changes occur. ^18^F-FDG-PET imaging of many cancers relies on their elevated glucose consumption, caused by high metabolic activity and an inefficient glycolysis producing lactate (known as Warburg effect). However, sensitivity of ^18^F-FDG-PET is generally lower for slowly growing cancers like prostate carcinoma or PDAC that lack a high glucose turnover. A recent study elaborated that supplemental ^18^F-FDG-PET can detect occult PDAC metastases not visible on ceCT, which in a clinical routine situation would have led to changes in staging and therapeutic strategy for a substantial proportion of these patients (6). The cancer specificity of ^18^F-FDG-PET is furthermore limited because activated macrophages, e.g., in inflammation, also show enhanced glucose consumption, which sometimes makes it difficult to accurately diagnose certain clinical scenarios, such as differentiating cancer from inflammatory lesions.

A higher sensitivity and specificity in cancer imaging can be achieved with radiopharmaceuticals that bind to specific cellular biomarkers for certain cancer types or subgroups. In recent years, this approach has revolutionized the clinical management of neuroendocrine tumors (NET) or prostate carcinoma (PCa), fostered by the availability of novel specific imaging agents. Particularly successful examples are ^68^Ga-DOTATOC (7, 8) or ^68^Ga-PSMA-11 (9, 10), which selectively bind to the somatostatin receptor 2 (SSTR2) or prostate-specific membrane antigen (PSMA), respectively, that are highly expressed by NET or PCa, respectively. Likewise, the transmembrane cell adhesion receptor αvβ6-integrin is frequently expressed in high density on the surface of many carcinoma cell types, such as various head-and-neck cancers, non-small cell lung cancer (NSCLC), and particularly PDAC (11). αvβ6-integrin activates transforming growth factor beta (TGF-β) which, in turn, promotes tumor growth and invasion, and furthermore leverages cancer immune escape by suppressing the antitumor activity of *T*-cells (12). Of note, other integrin subtypes, particularly αvβ3-integrin, have been extensively investigated for cancer diagnostics in the past, mainly in the context of targeting activation of endothelial cells for imaging of tumor angiogenesis. However, αvβ6-integrin is expressed by epithelial cells only, resulting in a substantially different scope of potential applications (13, 14). A particular advantage of using αvβ6-integrin as an imaging target is that it is only expressed at low levels by most adult human cell types. Similar to the aforementioned oncological targets SSTR2 and PSMA, addressing of αvβ6-integrin therefore has the potential to enable the specific, inflammation-insensitive imaging of PDAC. In 2019, Hausner et al. reported the preclinical development and first-in-human PET imaging of αvβ6-integrin with ^18^F-αvβ6-binding peptide in metastatic carcinoma, confirming its applicability to a broad spectrum of malignancies (15).

There is a high clinical need to improve both specificity and sensitivity in diagnosis of metastatic pancreatic cancer. For example, in trials comparing the efficacy of neoadjuvant vs. adjuvant therapy of pancreatic cancer, utilizing conventional imaging and laparoscopy, 25% of the 121 patients randomly assigned to immediate surgery were unable to undergo resection due to the presence of distant metastases or local non-resectability. Consequently, these patients underwent an unnecessary surgical procedure. In the preoperative arm, 72 out of 119 patients underwent resection. Among these, 37 achieved R0 resection, while the remaining 51 patients underwent resection in both arms, with and without neoadjuvant therapy (16). Despite the implementation of curative resection, the long-term patient survival rate remained between 30% and 40%. Even after intense adjuvant treatment with FOLFIRINOX, it was found that 30%–50% of patients metastasized within the first 12 and 24 months, respectively (17). Such data highlight the necessity to improve preoperative diagnostics to reduce unnecessary surgical intervention, to optimize neoadjuvant or surgical treatment according to the tumor stage, and to enhance the treatment outcomes for patients with advanced or metastatic tumors.

The diagnostic value of αvβ6-integrin targeted PET tracers in other tumor entities or other diseases with αvβ6-integrin expression, like NSCLC, idiopathic pulmonary fibrosis, and in lungs after SARS-COV2-Infections, has been highlighted in previous works (18). Radiotracers targeting αvβ6-integrin such as 18F-FP-R01-MG-F2 are currently being clinically tested. Recently, the radiopharmaceutical ^68^Ga-Trivehexin has been suggested as a specific PET imaging agent for PDAC (19). A recently published mini-review by Kimura et al. emphasized that ^68^Ga-Trivehexin performed particularly well in pancreatic cancer (20). Along these lines, we aimed to determine biokinetics, contrast with surrounding tissue and background, and parameters required for optimal image acquisition of ^68^Ga-Trivehexin.

## Materials and methods

### ^68^Ga-Trivehexin synthesis

Radiolabeling was performed by an automated procedure using a Modular-Lab module (Eckert & Ziegler, Berlin, Germany) with a ^68^Ge/^68^Ga-Generator (GalliaPharm® by Eckert & Ziegler AG, Berlin, Germany) in a laminar flow hood. In summary, a StrataXC cartridge combined radioactivity of two generators. The StrataXC cartridge was preconditioned with 5 ml of 0.2 M HCl (Merck suprapur®). After trapping the ^68^Ga activity, the StrataXC cartridge was rinsed with air, and the remaining activity was eluted with 800 µl of 0.06% HCl in acetone into the reaction vial. The vented reaction vial contained 45 µg of precursor, 150 µl 0.2 M sodium acetate (Merck suprapur®) buffer (pH 3.8–4.0) and 150 ml of absolute ethanol (Ph. Eur.) to reach a reaction pH of 2.0–2.5. After a reaction time of 5 min at 95°C, the reaction was quenched by adding water and the product was trapped onto a C18-cartridge (WAT023501, Waters) preconditioned with 1 ml EtOH and 3 ml H_2_O. Purification involved eluting the product with 2 ml EtOH:H_2_O directly through a sterile filter into the product vial and rinsing the cartridge with 8 ml NaCl 0.9% (B.Braun) again through the sterile filter for product dilution. The quality control release criteria (endotoxin level <5.00 EU/ml; radio-TLC > 95%; radio-HPLC > 95%; pH 4.0–8.0) were met for all radiosyntheses. Sterility was tested retrospectively after complete decay (3 days post-synthesis). The syntheses typically yielded 450 ± 45 MBq of the final product, starting from about 600 MBq. ^68^Ga-Trivehexin was administered in compliance with the German Medicinal Products Act (Arzneimittelgesetz §13 Abs. 2b) and with the approval of the responsible local regulatory authority.

### Patients

Between July 2021 and October 2022, a retrospective analysis was conducted on 44 patients (23 female, 21 male; mean age 62.6 years, range 37–82 years) who had undergone ^68^Ga-Trivehexin PET/CT (Table 1). One patient was examined three times. All patients provide written informed consent in advance. The data analysis received approval from the responsible local ethics committees (EK-242052023).

**Table 1:** List of pancreatic cancer patients (suspected or confirmed) who were examined using ^68^Ga-Trivehexin PET/CT.

Patient	Age	Dose [MBq]	Sex	Time [min]	CTx prior scan	Findings
1	59	139	M	61	No	Pancreatic cancer, liver metatasis
2	71	152	W	60	No	Pancreatic cancer
3	53	144	M	64	Yes	
4	60	96	W	60	Yes	Pancreatic cancer, bone metastasis
5	70	132	M	60	Yes	Pancreatic cancer
6	65	139	M	60	Yes	Pancreatic cancer
7	63	148	M	61	No	Pancreatic cancer
8	71	153	W	59	Yes	Pancreatic cancer
9	66	143	W	60	Yes	Pancreatic cancer
10	48	151	M	60	Yes	Pancreatic cancer, peritoneal carcinomatosis
11	72	145	W	60	No	Pancreatic cancer, lymph node metastasis, pulmonary metastasis
12	53	151	W	60	Yes	Lymph node metastasis
13	73	152	W	60	Yes	Pancreatic cancer
14	51	152	W	61	Yes	Pancreatic cancer, bone metastasis
15	63	160	M	60	Yes	Pancreatic cancer
16	48	130	W	60	No	Pancreatic cancer, liver metatasis, lymph node metastasis
17	82	123	W	60	Yes	Pancreatic cancer, liver metatasis, peritoneal carcinomatosis
18	49	154	M	60	Yes	
19	63	122	M	66	Yes	Pancreatic cancer, liver metatasis, lymph node metastasis
20	48	157	M	64	Yes	Pancreatic cancer, liver metatasis
21	69	94	M	60	Yes	Liver metatasis, peritoneal carcinomatosis, pleural carcinomatosis, soft tissue metastasis
22	71	111	M	55	No	Pancreatic cancer
23	50	146	M	60	Yes	Pancreatic cancer, lymph node metastasis, secondary tumor
24	74	143	W	60	Yes	Pancreatic cancer, liver metatasis, lymph node metastasis, peritoneal carcinomatosis
25	63	84	W	67	Yes	Pulmonary metastasis
26	58	93	M	71	No	Pancreatic cancer, lymph node metastasis
27	76	157	W	81	Yes	Liver metatasis
28^a^	42	125	M	63	No	Pancreatic cancer
29	71	158	W	60	Yes	Pancreatic cancer
30	73	150	M	60	Yes	Pancreatic cancer, secondary cancer
31	37	160	M	60	Yes	Pancreatic cancer, lymph node metastasis
32	69	140	W	56	No	Pancreatic cancer, liver metatasis, lymph node metastasis, peritoneal carcinomatosis
33	66	156	W	60	Yes	Pancreatic cancer
34	67	128	W	85	Yes	Pancreatic cancer, liver metatasis, peritoneal carcinomatosis
35	59	146	W	60	No	Pancreatic cancer
36	59	147	W	60	No	Liver metatasis
37	71	149	M	60	Yes	Pancreatic cancer, liver metatasis, secondary tumor
38	58	114	M	67	Yes	
39	52	148	M	60	No	Pancreatic cancer, liver metatasis, lymph node metastasis
40	80	150	M	60	No	Pancreatic cancer, splenic metastasis
41	72	128	W	66	No	Pancreatic cancer, lymph node metastasis
42	66	142	W	60	No	Pancreatic cancer
43	52	147	W	60	Yes	Pancreatic cancer
44	71	157	W	60	No	Pancreatic cancer

^a^ Patient with three examinations.

### Imaging procedure

All PET measurements were conducted using a digital Siemens Biograph Vision 600 scanner (Siemens Medical Solutions, Erlangen, Germany). A low-dose CT (120 kVp, 78 mAs, spiral pitch factor of 1.5, 512 × 512 matrix with a pixel distance of 0.98 mm) without contrast was performed for attenuation correction before the subsequent PET scan. The 3D PET data was obtained in list mode using continuous bed motion. Patients received intravenous administration of 139 MBq (range: 84–160 MBq) of ^68^Ga-Trivehexin, molar activity 31 ± 8 MBq/nmol, mass dose 4.7 ± 1.6 nmol, with no adverse effects associated with the radiotracer application. For dynamic PET imaging, ^68^Ga-Trivehexin was injected intravenously over 20 s. List mode acquisition commenced at the start of ^68^Ga-Trivehexin infusion, covering 45 min in one bed position. Subsequent sequential PET frames (6 × 30 s, 7 × 60 s, 7 × 300 s) were reconstructed. After dynamic PET and 55–85 min after ^68^Ga-Trivehexin injection, static ^68^Ga-Trivehexin-PET/CT scans were acquired with five bed positions assessing the whole body, aligning with the exposure time of ^18^F-FDG.

PET images were reconstructed following our standard routine using an ordered subset expectation maximization (OSEM) 3D iterative reconstruction algorithm with 4 iterations and 5 subsets (4i5s), applying point spread function, and time of flight with an image matrix size of 440 × 440, resulting in a voxel distance of (1.65 × 1.65 × 1.65) mm^3^. Reconstructions were performed with attenuation and relative scatter correction and no post-filtering.

### Image analysis

Data were analyzed by a certified nuclear medicine physician and radiologist using dedicated workstation and software (Syngo MMWP and Syngo TrueD, Siemens Medical Solutions, Erlangen, Germany). For quantitative analysis of tracer uptake, a SUV-based analysis in the respective lesions was performed. For static PET scans, SUVmean and SUVmax of pancreatic cancer and metastases were measured using a volume of interest (VOI) technique. VOIs were defined by an automatic isocontour with a cutoff at 50% of SUVmax. The tumor-to-liver ratio (TLR) was calculated. To obtain the SUVmax for the liver, three VOIs (>1.5 cm^3^) were drawn from the right hepatic lobe and averaged (SUVavg), respectively. TLR was calculated as TLR = SUVmax/SUVavg. For dynamic PET imaging analysis, VOIs of pancreatic cancer (SUV pancreatic cancer), pancreas (SUV pancreas background), stomach wall (SUV stomach wall), blood pool (SUV blood pool), liver (SUV liver), as well as liver metastasis (SUV liver metastasis) and lymph node metastasis (SUV lymph node metastasis) were drawn and applied to the entire dynamic dataset.

### Collection of tissue samples and immunohistochemistry

Formalin-fixed paraffin-embedded (FFPE) tissue blocks of pancreatic ductal adenocarcinomas (PDAC) from the archive of the Institute of Pathology of the University hospital Dresden were cut into 2 µm sections using a rotating microtome. Human tissue was immunohistochemically examined using an anti-human Integrin β6 (ITGB6) mouse monoclonal antibody [clone 442.5C4] (#407317, dilution 1:100, Merck Millipore, Burlington, Massachusetts, USA). Immunohistochemistry (IHC) was performed on an autostainer (Bond RX^m^, Leica Biosystems, Wetzlar, Germany). Antigen retrieval (AR) was achieved by using enzyme pretreatment (Bond^TM^ Enzyme Pretreatment E1, #AR9551, Leica Biosystems, Wetzlar, Germany) for 5 min. Finally, antibody binding was visualized by using a brown chromogen [3,3’-diaminobenzidine (DAB)] (#DS9800, Bond Polymer Refine Detection, Leica Biosystems, Wetzlar, Germany). Slides were scanned in 40× magnification using a bright field slide scanner (Leica AT2) and subsequently digitally evaluated (Aperio ImageScope ×64).

Membranous staining intensity of the majority of tumor cells on each slide (score 0–3) and the frequency of β6-integrin positive tumor cell membranes (in%) were assessed per whole tissue section. A combined final score was then calculated by multiplying the factors score × frequency [histological score modified according to Sipos et al. (21)].

### Statistical analysis

Data were retrospectively collected from patients undergoing clinical examinations. The dataset included patient demographics, examination details, and various medical findings. All statistical analyses were performed using Python (version 3.8.10) in combination with Pandas (version 1.2.4) and Statsmodels (version 0.12.2). Basic statistical measures, including mean, median, standard deviation, minimum, and maximum were computed for age, dose, and time until examination.

For static PET parameters, such as SUVmax of pancreatic cancer, metastases, and TLR, comprehensive data were collected. Box plots were generated for a visual assessment of data distribution, outliers, and spread. A one-way ANOVA test was conducted to ascertain statistically significance differences among group means. Upon identifying significant differences (*p* < 0.05) in the ANOVA test, a Tukey HSD post-hoc test was applied to identify which specific groups’ means (compared pairwise) were different. To analyze tumor metastases intensity metrics across subgroups with and without previous chemotherapy, we conducted a comprehensive analysis on a dataset comprising SUVmax and TLR measurements. Subsequently, *t*-tests for independent samples were performed to assess the statistical significance of observed differences. The threshold for statistical significance was set at *p* < 0.05. Cases with insufficient data for robust analysis were flagged and excluded from further evaluation.

For dynamic PET data, measurements (SUVmax and SUVmean) were taken at specific time intervals post-injection, varying from short intervals at the beginning to longer intervals towards the end. Each interval comprised multiple measurements for every organ and tissue (*n* = 11, *n* (liver metastases) = 13, *n* (lymph node metastases) = 13). Averaging over time within each interval yielded interval-specific SUVs. Data were presented for SUV measurements over time (*t*). The primary objective was to identify the time point where the SUV of the tumor (either mean or max) peaked while maximizing the difference between the tumor's SUV and that of other regions. The central aim was to derive ratios of SUVmax of pancreatic cancer to background tissue (tumor-to-background ratio, TBR), specifically, tumor-to-pancreas, tumor-to-liver, tumor-to-blood pool, tumor-to-stomach wall, and to determine the time point for each ratio to peak. Therefore, we employed the following mathematical formulation:

$$t_{\max}(R)=\arg_{\;\;\;\;t}^{\max}R(t)$$

Where:

• $t_{\max}(R)$ indicates the time when the ratio *R* achieves its maximum value.
• $\arg_{\;\;\;\;t}^{\max}$ designates the time *t* at which the ratio *R* is maximized.
• R(t) gives the value of the ratio *R* at a specific time *t*.

## Results

### Patients

In our dataset of 44 patients, the mean age was approximately 62.6 years, with a range from 37 to 82 years (Table 1). The gender distribution was nearly equal, with 52% being female and 48% male.

The average administered activity was 139 MBq, ranging from 84 to 160 MBq. Static acquisitions were started between 55 and 85 min p.i. (avg. 62 min).

**Figure 1:**
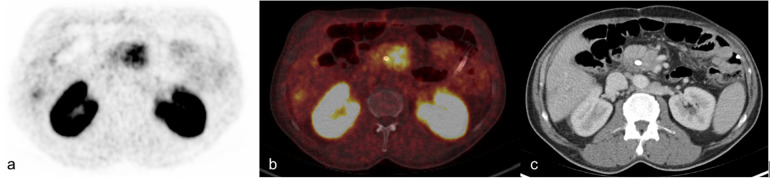
Primary staging of 59-year-old man with pancreatic cancer and simultaneous liver metastases (139 MBq ^68^Ga-trivehexin, 61 min p.i). **(a)** PET image **(b)** fused image with correlating ceCT, **(c)** contrast-enhanced CT in transversal plane.

**Figure 2:**
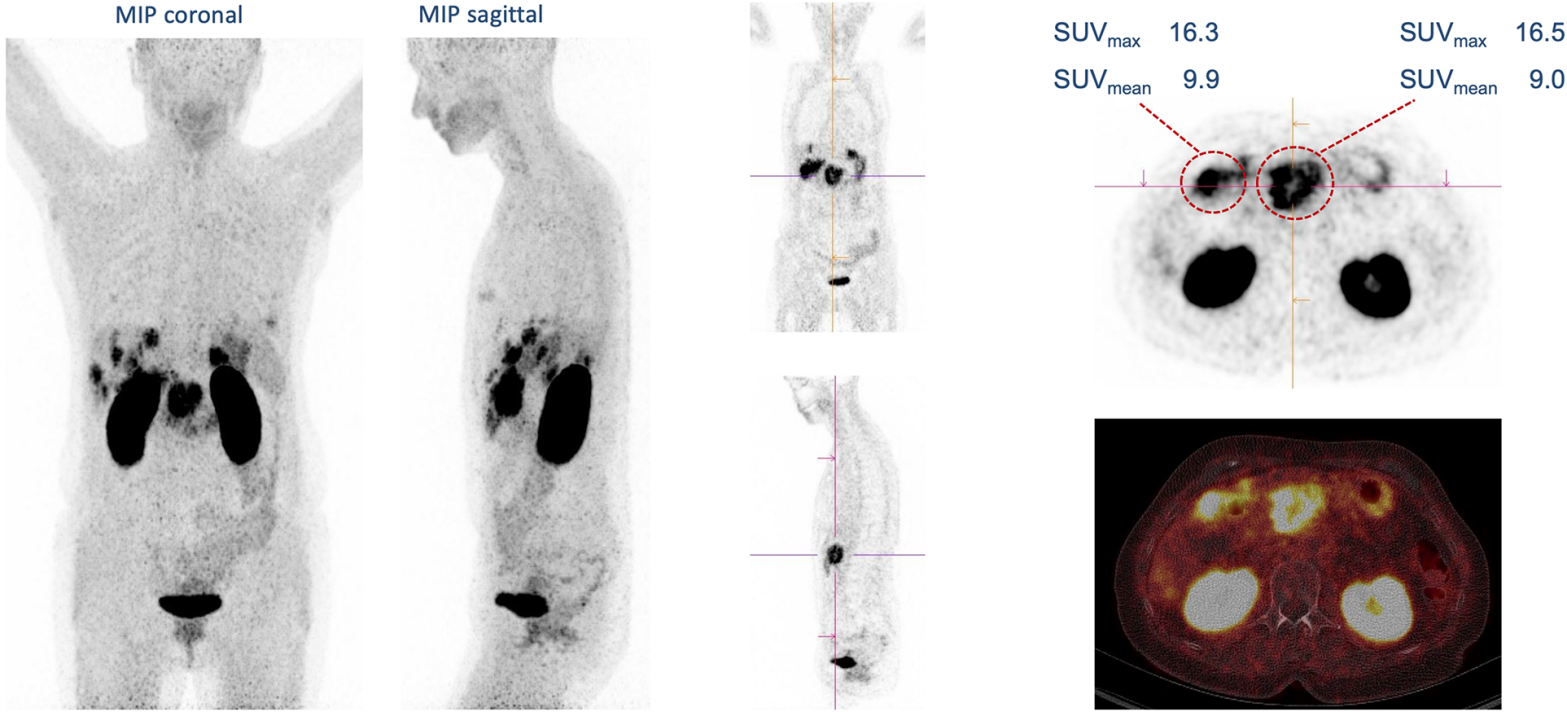
Primary staging of 67-year-old man with pancreatic cancer, simultaneous liver metastases and peritoneal carcinomatosis (128 MBq ^68^Ga-trivehexin, 85 min p.i.).

At the commencement of the investigation, a collective of 23 patients were identified with pre-existing distant metastases, of which 10 presented simultaneous distant metastatic and lymph node metastases. One patient exhibited only lymph node metastases. The predominant pattern revealed solitary pancreatic cancer in 17 patients. A collective of 13 patients exhibited liver metastases (Figure 1), while six had peritoneal carcinomatosis (Figure 2). Less frequent observations included secondary neoplasms, osseous metastases, pulmonary metastases, splenic metastases, pleural carcinomatosis and metastases in soft tissues, among other observations. Furthermore, there have been patients with liver metastases occurring concurrently with lymph node metastases and pancreatic cancer, but less frequently.

**Figure 3:**
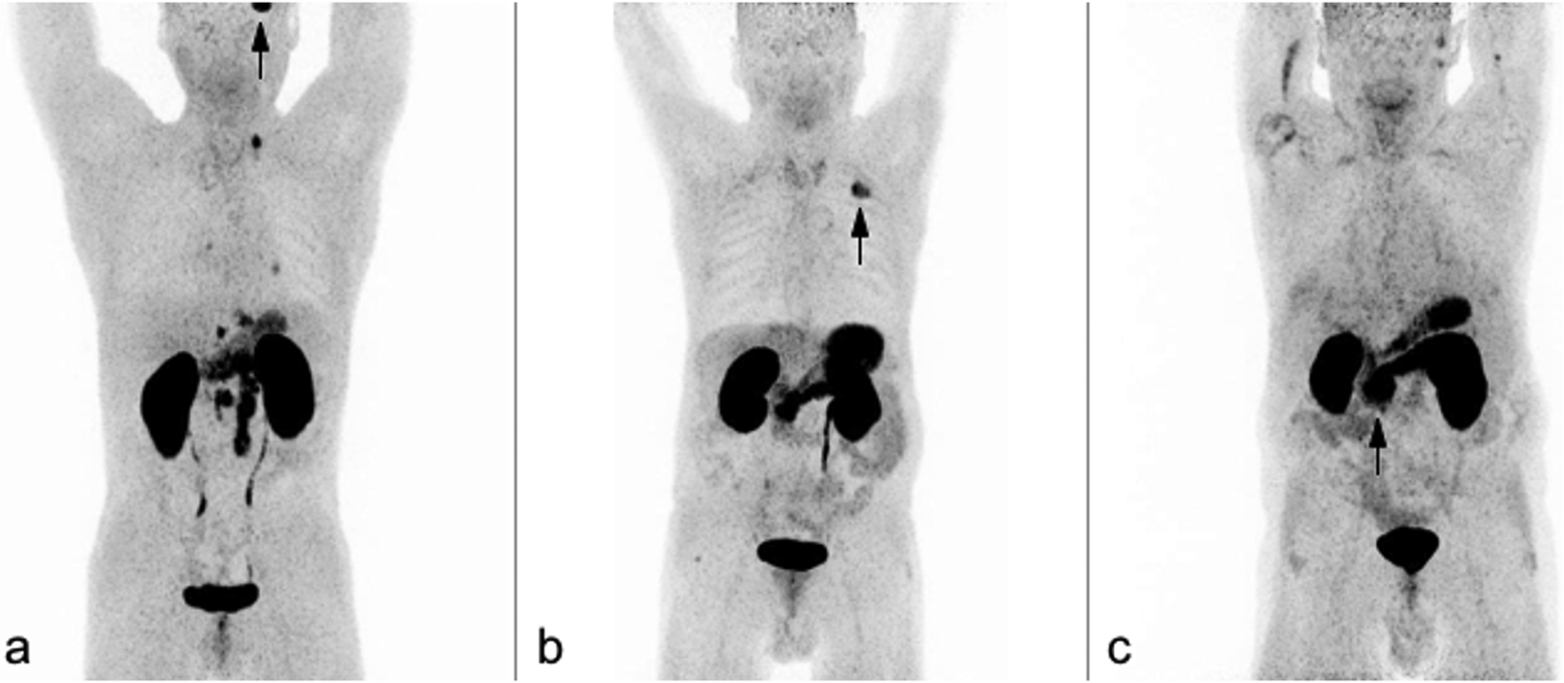
Coronary maximum intensity projections (MIP) of ^68^Ga-trivehexin PET scans. **(a)** 50-year-old man with lymphogenically metastasized pancreatic cancer, and supratentorial metastasis of squamous cell carcinoma of the tonsil (black arrow). **(b)** 71 squamous cell carcinoma of the left upper lobe (black arrow). **(c)** 73-year-old man with pancreatic cancer simultaneously presents with a moderately differentiated extrahepatic cholangiocarcinoma of pancreatobiliary subtype (black arrow) with high uptake throughout the entire pancreatic parenchyma.

Uptake of ^68^Ga-Trivehexin in non-metastatic organs was most frequently observed in the uterus, tissues recently tangentially affected by surgery or intervention, and less frequently in non-enlarged lymph nodes (Table 2). Notably, intense uptake was observed in a supratentorial brain metastasis (left cerebellopontine angle) of a squamous cell carcinoma of the tonsil (SUVmax 18.5), in a simultaneous extrahepatic cholangiocarcinoma of pancreatobiliary subtype (SUVmax 21.5) with intense uptake of the entire pancreatic parenchyma, and a histologically confirmed squamous cell carcinoma of the left upper lobe (SUVmax 8.8) as seen in Figure 3. It is important to note that these are cases of pancreatic cancer with concomitant metachronous tonsillar carcinoma and its metastases. Specifically, it was a tonsillar carcinoma with cervical metastases on the right with subsequent surgical resection and RCTx.

**Table 2:** ^68^Ga-Trivehexin uptake (SUVmax, SUVmean) in histologically confirmed secondary malignancies and non-metastatic organs.

Patient	Findings	SUVmax	SUVmean
1	Fractured rip	5.5	3.3
1	Axillary lymph node	12.0	7.8
3	Thyroid nodule	5.9	3.7
8	Pneumonia	4.3	2.6
9	Uterus	10.7	5.8
11	Pulmonary nodulus located in segment 8/9 (r)	2.9	1.8
12	Uterus	6.3	3.6
12	Indistinct hepatic lesion located in segment II	4.7	2.8
14	Hilar lymph node	4.4	2.9
16	Uterus	7.3	4.8
16	Mamma	2.8	1.8
17	Uterus	6.7	4.0
23	Hilar lymph node	5.2	2.5
23^a^	Supratentorial brain metastasis (left cerebellopontine angle) of a squamous cell carcinoma of the tonsil	18.5	11.1
25	Mamma	4.3	2.7
25	Stomach after subtotal gastric resection with reconstruction (Roux-Y anastomosis)	9.0	5.1
25	Acromioclavicular joint	3.6	2.3
26	Hematoma in the upper right abdomen	9.3	5.1
29	Axillary lymph node	4.3	2.6
30^a^	Simultaneous bile duct carcinoma	21.5	12.2
33	Uterus	8.2	4.7
36	Port	4.5	2.4
37^a^	Squamous cell carcinoma of the left upper lobe	8.8	5.3
38	Fractured rib	7.0	4.3
40	Prostate	12.5	7.6

^a^ Indicates proven secondary malignancies.

### Quantitative analysis

**Figure 4:**
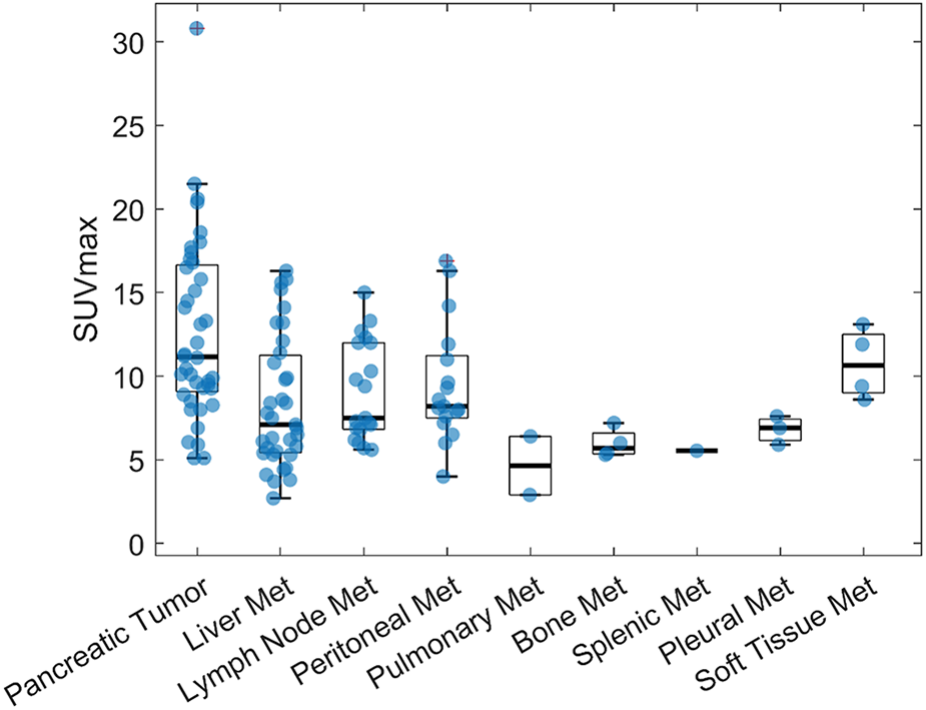
^68^Ga-Trivehexin SUVmax for pancreatic cancer primaries and metastases.

In our dataset, the frequency for each category was as follows: pancreatic tumor (*n* = 40), liver metastases (*n* = 39), lymph node metastases (*n* = 21), peritoneal carcinomatosis (*n* = 17), pulmonary metastases (*n* = 2), bone metastases (*n* = 4), splenic metastasis (*n* = 1), pleural carcinomatosis (*n* = 3) and soft tissue metastases (*n* = 4) (Figure 4). Preliminary analysis indicated variability in SUVmax and TLR measurements across each category as seen in Table 3. A one-way ANOVA test was conducted (F-value = 4.55, *p* = 7.1193 × 10^−5^), demonstrating significant differences in the means across groups (*p* < 0.05). This warranted the execution of further post-hoc tests. Tukey post-hoc analysis revealed a significant difference (*p* < .001) between the groups SUVmax (liver metastasis) and SUVmax (pancreatic cancer) [4.74, 95%-CI (1.74, 7.75)], SUVmax (lymph nodes metastasis) and SUVmax (pancreatic cancer) [4.07, 95%-CI (0.47, 7.67)], TLR (liver metastasis) and TLR (pancreatic cancer) [1.82, 95%-CI (0.83, 2.80)], TLR (pancreatic cancer) and TLR (peritoneal carcinomatosis) [−1.88, 95%-CI (−3.15, −0.61)], and TLR (pancreatic cancer) and TLR (pleural carcinomatosis) [−2.79, 95%-CI (−5.42, −0.18)].

**Table 3:** SUVmax and TLR for pancreatic cancer and metastases.

	Avg.	Std	Min	Max	Range
SUVmax pancreatic cancer	12.6	5.4	5.1	30.8	25.7
SUVmax liver metastasis	7.9	4.0	2.7	16.3	13.6
SUVmax lymph node metastasis	8.6	3.3	2.5	15.0	12.5
SUVmax peritoneal carcinomatosis	9.5	3.5	4.0	16.9	12.9
SUVmax pulmonary metastasis	4.7	2.5	2.9	6.4	3.5
SUVmax bone metastasis	6.0	0.9	5.3	7.2	1.9
SUVmax splenic metastasis	5.5		5.5	5.5	0.0
SUVmax pleural carcinomatosis	6.8	0.9	5.9	7.6	1.7
SUVmax soft tissue metastasis	10.8	2.1	8.6	13.1	4.5
TLR pancreatic cancer	4.9	1.7	1.7	9.4	7.7
TLR liver metastasis	3.0	1.3	1.1	5.9	4.8
TLR lymph node metastasis	3.9	1.5	1.7	8.0	6.2
TLR peritoneal carcinomatosis	3.0	0.7	1.4	3.9	2.5
TLR pulmonary metastasis	2.2	1.0	1.5	3.0	1.5
TLR bone metastasis	3.7	0.8	2.7	4.6	1.9
TLR splenic metastasis	1.8		1.8	1.8	0.0
TLR pleural carcinomatosis	2.1	0.3	1.8	2.3	0.5
TLR soft tissue metastasis	3.3	0.6	2.6	4.0	1.4

avg, average; std, standard deviation; min, minimum; max, maximum.

In the examination of tumor metastases intensity across two subgroups, delineated as with or without CTx prior scan, various metrics were assessed for their significance. Notably, the TLR (pancreatic cancer) and TLR (peritoneal carcinomatosis) metric revealed a significant difference. Other metrics, such as SUVmax (pancreatic cancer) and SUVmax (peritoneal carcinomatosis), approached significance but did not surpass the commonly accepted threshold of *p* < 0.05 as seen in Table 4. Several metrics could not be adequately assessed due to insufficient data.

**Table 4:** Comparison of SUVmax and TLR for pancreatic cancer and metastases for subgroups with and without chemotherapy previous examination.

Metric	*p*-value	Avg. (no therapy)	Std (no therapy)	Avg. (therapy)	Std (therapy)
SUVmax pancreatic cancer	0.08	14.6	4.4	11.5	5.7
SUVmax liver metastasis	0.51	7.9	4.1	8.8	3.8
SUVmax lymph node metastasis	0.36	8.3	2.5	9.6	3.2
SUVmax peritoneal carcinomatosis	0.07	5.3	1.8	10.1	3.3
SUVmax pulmonary metastasis	Insufficient data	2.9		6.4	
SUVmax bone metastasis	Insufficient data			6.0	0.9
SUVmax splenic metastasis	Insufficient data	5.5			
SUVmax pleural carcinomatosis	Insufficient data			6.8	0.9
SUVmax soft tissue metastasis	Insufficient data			10.8	2.1
TLR pancreatic cancer	0.02	5.6	1.6	4.4	1.6
TLR liver metastasis	0.53	3.2	1.4	2.9	1.2
TLR lymph node metastasis	0.47	4.4	1.4	3.8	1.7
TLR peritoneal carcinomatosis	0.02	1.9	0.6	3.1	0.6
TLR pulmonary metastasis	Insufficient data	1.5		3.0	
TLR bone metastasis	Insufficient data			3.7	0.8
TLR splenic metastasis	Insufficient data	1.8			
TLR pleural carcinomatosis	Insufficient data			2.1	0.3
TLR soft tissue metastasis	Insufficient data			3.3	0.6

avg, average; std, standard deviation.

### Dynamic PET imaging

**Figure 5:**
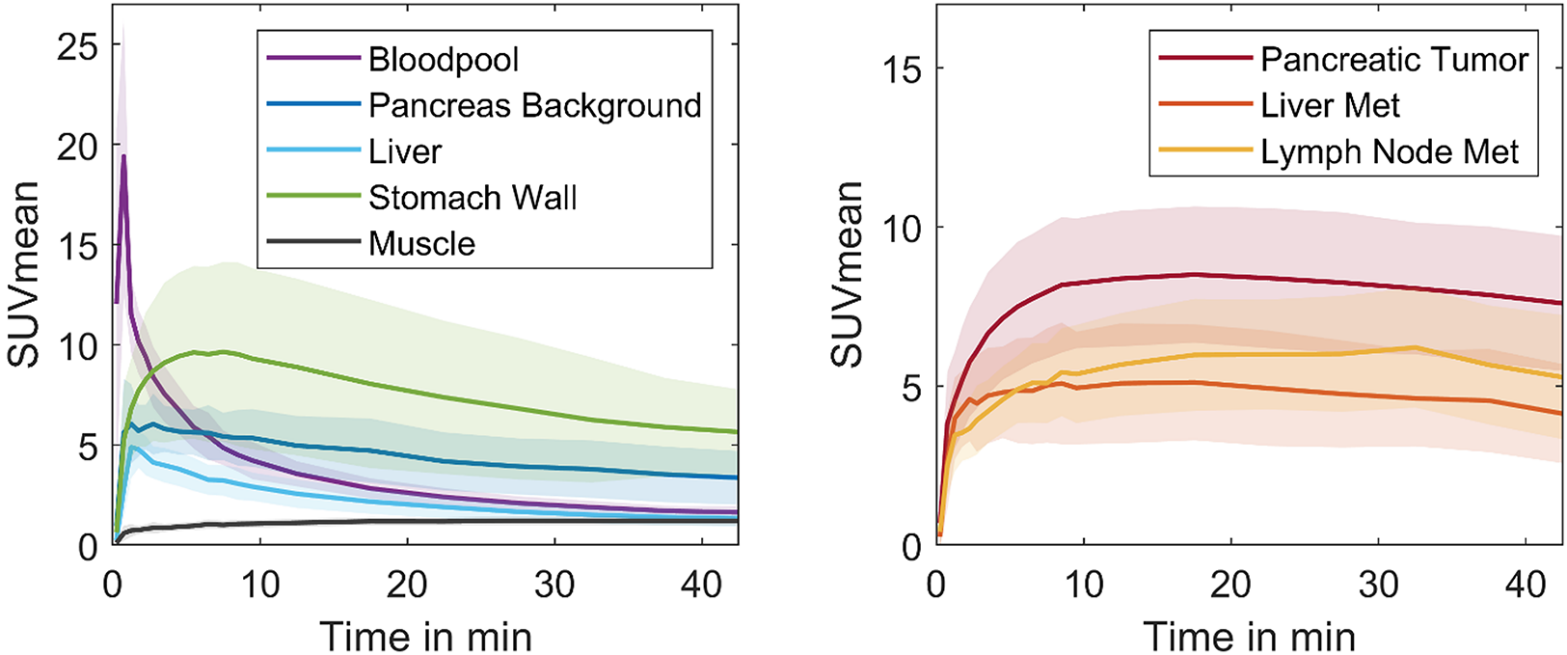
SUVmean-time plots for physiology parameters (left) and pancreatic cancer primaries and metastases (right), derived from 45 min dynamic PET scans of 11 patients.

During dynamic PET imaging, both liver and pancreatic tissues showed a temporary increase in activity, with the SUVmean of the pancreas surpassing that of the liver (Figure 5). After reaching a maximum, the SUVmean of both organs steadily decreased. Pancreatic cancers exhibited delayed uptake compared to the surrounding normal pancreatic tissue. Over the dynamic scanning process, there was an accumulation of ^68^Ga-Trivehexin within the tumor, ultimately leading to the formation of a plateau, following only a slight reduction in the SUVmean. Liver metastases and lymph node metastases had a comparable pattern to pancreatic malignancies.

**Figure 6:**
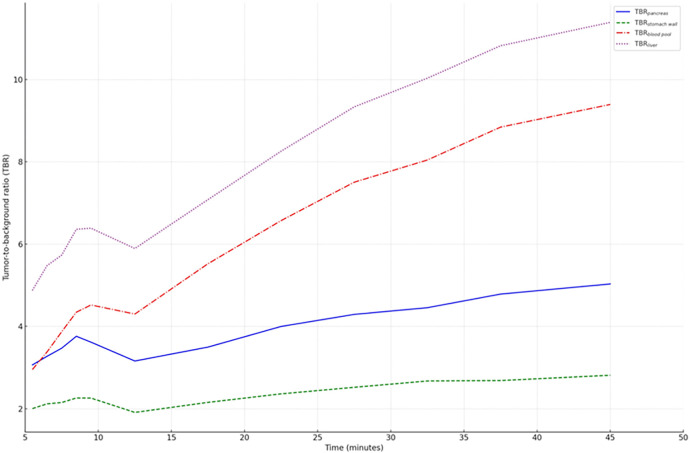
Time-dependent tumor-to-background ratios (TBR) for various organs. The highest TBR values were observed at 45 min p.i., suggesting that this point offers optimal imaging contrast for distinguishing tumors from background tissue.

Interestingly, the rate and magnitude of activity uptake in the stomach wall surpasses that observed in pancreatic cancer during the initial phase of the dynamic scan. After approx. 10 min, the SUVmean of the stomach wall reached a plateau and subsequently decreased. After 17.5 min, the SUVmean of pancreatic cancer exceeded that of the stomach wall. The difference in SUVmean values between pancreatic cancer and other organs, including the stomach wall, pancreas background, and liver, gradually increased throughout the dynamic measurement until completion. The analysis pinpointed the optimal imaging time for maximum contrast between pancreatic cancer and surrounding tissues (TBR) to be 45 min following radiotracer injection (Figure 6).

### Histology and ITGB6 immunohistochemistry

**Figure 7:**
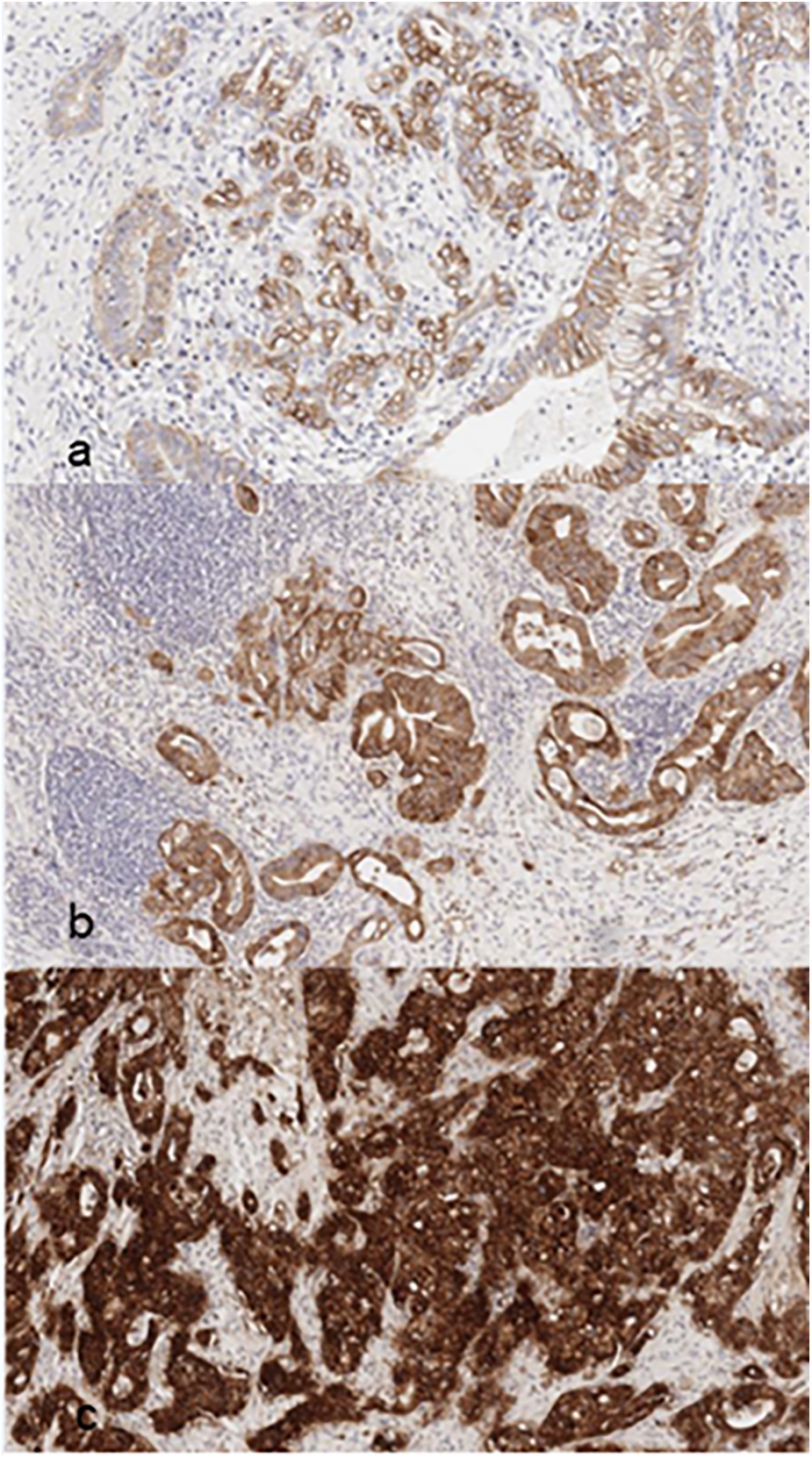
Examples for ITGB6 (β-6-integrin) immunohistochemistry (IHC) with corresponding PDAC-scores **(a)** PDAC-score 1; **(b)** PDAC-score 2; **(c)** PDAC-score 3.

23 primary pancreatic ductal adenocarcinoma (PDAC) resection specimens and two distant metastases (liver, lung) contained a sufficient amount of tumor cells for immunohistochemical evaluation of membranous ITGB6 expression. All PDACs showed clear positivity for membranous ITGB6. The examined samples demonstrated varying degrees of ITGB6 positivity: 3/25 (12%) scored 1, 9/25 (36%) scored 2, and 13/25 (52%) scored 3 (for examples see Figure 7).

## Discussion

The objective of our analysis was to elucidate the biokinetics of an αvβ6-integrin targeted PET radiopharmaceutical, ^68^Ga-Trivehexin, in the context of PET imaging patients diagnosed with pancreatic ductal adenocarcinoma (PDAC). We also explored image contrast (particularly comparing patients with and without prior chemotherapy) and identified the essential parameters for effective imaging. The aggressiveness of this cancer and the necessity of early detection are highlighted by the fact that 23 patients in our study had already developed distant metastases. Our findings indicated that ^68^Ga-Trivehexin is well-suited for imaging αvβ6-integrin expression in pancreatic cancer. No adverse events were recorded. Immunohistochemical findings showed consistent αvβ6-integrin expression in all examined specimens. A more detailed analysis, correlating malignant lesions precisely with immunohistochemistry, is recommended for future studies. The primary tumor as well as the metastases were visualized with a high tumor-to-background ratio. Even after chemotherapy, the primary tumor, its lymph node, and hematogenous metastases remained detectable in several patients. This fact can at least be substantiated by the publication by Vujik et al. (22). This study demonstrates that αvβ6 expression remains present in PDAC following chemotherapy, indicating that the target is not downregulated by the treatment. The results obtained with Trivehexin further corroborate these findings. The quantitative analysis revealed significant differences in tracer uptake between primary tumor and metastases, with both exhibiting a high radiotracer accumulation. Significantly different levels of uptake were observed in the groups SUVmax (liver metastasis) and SUVmax (pancreatic cancer), SUVmax (lymph node metastasis) and SUVmax (pancreatic cancer), and TLR (liver metastasis) and TLR (pancreatic cancer). These differences imply a stronger affinity of the tracer for primary tumors compared to metastatic lesions. This suggests that αvβ6 expression is more pronounced in the primary tumors than in the metastases. However, this would need to be investigated in more detail in further studies. Furthermore, the influence of chemotherapy of tracer uptake was evident when comparing SUV across subgroups with and without chemotherapy.

Consequently, it is necessary to consider a patient's medical history when interpreting imaging data. In addition, αvβ6-integrin expression can be increased in fibrotic diseases, including idiopathic pulmonary and renal fibrosis (23). While this points at a potential utility of ^68^Ga-Trivehexin for imaging of fibrosis, the unambiguous identification of metastases in patients with fibrotic disease might be difficult. We also made observations and findings that we could not adequately explain. For example, in one patient, there was homogenous ^68^Ga-Trivehexin-uptake throughout the entire pancreas parenchyma, although morphological abnormalities were limited to the pancreas head. Based on our present experience, we hold the view that in such cases of doubt, the additional acquisition of ceCT could be advised.

Our dynamic PET data showed temporal variation in the uptake of the tracer in different tissues, such as the primary tumor itself and the stomach. Among others, we identified two distinct kinetic types in pancreatic cancers with αvβ6-integrin expression. One subtype is characterized by an early peak in the time activity curve followed by a gradual decline (*n* = 10). The second subtype exhibits a pattern of accumulation followed by a peak and subsequent horizontal progression. The first subtype may represent lower-grade tumor variants (*n* = 1). Further investigation of this appears to be warranted. In contrast, other organs show an initial spike followed by a subsequent decline. At the beginning of the dynamic scan, uptake in the gastric wall is rapid and even more intense than in the pancreatic cancer. This can be challenging in accurately identifying locoregional lymph node metastases due to the proximity of the gastric wall and pancreas. Therefore, the timing of imaging plays an important role in maximizing the contrast between tumor and background. The dynamic measurements were performed for a duration of 45 min. Here, the TBR showed the best contrast after the maximum recording time (45 min). This time frame was considered ideal for the start of the investigation, but the future evolution of the TBR cannot be accurately predicted. However, based on the above considerations, we believe that starting the examination 30–60 min p.i. is sufficient to accurately delineate the tumor from the surrounding tissue with a reasonably high TBR. TBR of the gastric wall increases minimally with time and the gastric wall can be well delineated in all patients with this nonspecific enhancement. Additional ceCT is useful for further delineation and is usually performed anyway, at least in primary staging, to assess local vascular infiltration and thus operability.

Taken together, this work highlights the clinical use of ^68^Ga-Trivehexin PET/CT for a more sensitive and selective detection of pancreatic cancer and its metastases, potentially enabling better diagnostics for an improved patient management. Although our data analysis is limited by its retrospective design and the relatively small patient cohort, we have seen clear indications for a better detection of occult metastatic disease as compared to the standard of care, ceCT and MRI. We therefore consider it urgently necessary to generate the respective clinical evidence in prospective clinical studies. Furthermore, it must be mentioned that other molecular targets have also been successfully used for imaging pancreatic and other cancers, of which fibroblast activation protein (FAP) is probably the most popular today and is widely imaged with ^68^Ga labeled FAP inhibitors (^68^Ga-FAPI). There is however a fundamental difference in the underlying biochemistry because αvβ6-integrin is expressed by the cancer cells, whereas FAP is expressed by cancer-associated fibroblasts (CAFs), and thus its presence is essentially restricted to the stroma. The diagnostic and prognostic values of both approaches are thus difficult to assess. A comparison of the SUVmax values measured for PDAC primaries after approximately 1 h for ^68^Ga-FAPI [13.4 ± 5.5; (24)] and ^68^Ga-Trivehexin (12.6 ± 5.4; this work) indicates a comparable performance, but more detailed studies appear to be necessary to come to a conclusion.

## Conclusion

^68^Ga-Trivehexin demonstrated high suitability for specific imaging of αvβ6-integrin expression in pancreatic cancer and metastases. To enhance tumor-background contrast, it is advisable to perform PET/CT at later time points. Our 45 min dynamic data indicated that the highest TBR was achieved at the end of data acquisition. A practical compromise is to acquire PET data 30–60 min p.i., followed by complementary diagnostic CT. This approach facilitates accurate detection of lymphogenic metastases between the stomach and pancreas, as well as recurrences along the arteries in the upper. Considering the aforementioned parameters, ^68^Ga-Trivehexin may adopt a significant role in future αvβ6-integrin directed theranostics.

## Data Availability

The original contributions presented in the study are included in the article/Supplementary Material, further inquiries can be directed to the corresponding authors.
